# Ultrasound-Guided Fine-Needle Aspiration Biopsy in Unselected Consecutive Patients with Thyroid Nodules

**DOI:** 10.5402/2011/284837

**Published:** 2011-07-14

**Authors:** Zdravko A. Kamenov, Vera N. Karamfilova, Georgi N. Chavrakov

**Affiliations:** Clinic of Endocrinology, Alexandrovska University Hospital, Medical University Sofia, 1 Georgi Sofiiski Street, Sofia 1431, Bulgaria

## Abstract

The objective was to analyze the results of UG-FNAB, performed in unselected consecutive patients with thyroid nodules. *Methods*. The UG-FNAB records were analyzed in this retrospective study. Indication for biopsy was the presence of at least one nodule detected by ultrasound. *Results*. 330 patients at mean age ± SD 48.4 ± 11.2 years; women/men = 12.8/1 were analyzed. From the total 596 nodules found 546 (91.6%) were investigated with 1231 punctures (2.3 per nodule and 3.7 per patient). Benign solitary nodules had 42.7%, multinodular goiter (MNG) 44.8%, inconclusive 4.8%, and others 2.1% and malignant nodules 5.5% of the patients (6.6% of solitary and 5.1% of MNG patients). The risk for a separate nodule in MNG to be malignant was 2.7%. *Conclusions*. UG-FNAB is a safe and reliable diagnostic approach for thyroid nodules. It is the method of choice for hypo- and isoechoic not purely cystic solitary nodules, regardless of the nodule size. In MNG, its positive predictive value and diagnostic accuracy are lower. The final decision for regular US monitoring, UG-FNAB of the dominant nodule, multipuncture UG-FNAB or surgical exploration is one of complex appraisal. We consider UG-FNAB appropriate for most nodules in MNG, according to the above mentioned criteria.

## 1. Introduction

Differentiated thyroid cancer is among the most rapidly increasing types of cancers with an incidence that has doubled in the past 15 years [1]. Ultrasonography (US) is the commonly used imaging technique for the thyroid gland. However, noninvasive differentiating benign from malignant lesions with clinically certainty is impossible even with modern equipment used by experienced specialists. A predominantly solid nodule, hypoechogenicity, microcalcification, ill-defined margins, intranodular vascularity, and taller-than-wide shape have all been associated with increased risk of malignancy, but no single US characteristic is sufficiently sensitive or specific to exclude or diagnose malignancy by itself. However, the use of combinations of US characteristics to stratify nodules into high- and low-risk for malignancy appears a promising strategy. [2]. 

More attention is paid to the vascular investigations. Color flow Doppler sonography (CFDS) and power Doppler are promising noninvasive methods. Some studies did not show any improvement in diagnostic accuracy and malignancy-predictive value for CFDS [3–6], but others suggested that Doppler is helpful [7–11], especially when using the second generation ultrasonographic contrast agents [12]. Recently, Sancak et al. compared CFDS and microvessel density determined by CD34-antibody staining and concluded that even the assessment of only the intranodular vascularization detected by CFDS does not result in a better discrimination of benign and malignant nodules nor does it give a better correlation with the histologic density of small vessels and microvessels. The authors also suggested that power Doppler technique may be helpful to show small vessels [13]. Moon et al. in a retrospective study using power Doppler ultrasonography reported that vascularity itself or a combination of vascularity and gray-scale US features was not as useful as the use of suspicious gray-scale US features alone for predicting thyroid malignancy [14]. While occasionally useful in selecting nodules for ultrasound-guided fine-needle aspiration biopsy (UG-FNA), color Doppler US should not be considered a requirement for the selection of nodules for sampling [15]. 

Fine-needle aspiration biopsy (FNAB) is routinely used to evaluate nodular thyroid disease. But the results of blind FNAB are often inconclusive, especially in cystic or mixed nodules. A combination of both methods by-UG-FNAB increases the diagnostic power of each of these methods (ultrasound and FNAB) and is costeffective as a first-line diagnostic procedure in patients with thyroid nodules [16–20]. Although broadly recognized as a method of choice for thyroid nodules, opinions still differ as to its use for other indications [1].

Thyroid nodules are estimated to be present in 20% or more of adults who are screened by routine thyroid echography [20]. The reported prevalence of nodules differs according to the population studied, with the most important determinants being age and iodine status. The prevalence of malignant lesions also varies. Most studies using the US technique are based on “retrograde analysis”; that is, they work back from morphologic results and analyze the coincidence with the numbers of lesions already diagnosed by cytology, FNAB and ultrasound. Specimens are usually preselected from the ultrasound and clinically suspicious lesions. Studies that evaluate the malignancy potential of every nodule either solitary or in the multinodular goiter (MNG) are rare. A recent consensus statement about the management of thyroid nodules detected at US [15] identified several important unanswered questions that merit future research. One of them is: In a patient with multiple nodules, which and how many nodules should undergo UG-FNAB?

**The Aim**

of this study was to analyze the results of UG-FNAB from 330 unselected consecutive patients with thyroid nodules.

## 2. Patients and Methods

The observational period in this retrospective study was from 1997 to 2006, with most biopsies (86.4%) taken between 2001 and 2006. Of the participants 92% attended the outpatients' department of a general endocrine clinic and 8% were hospitalized patients. The indication for biopsy was the presence of at least one solid or mixed nodule detected by ultrasound examination. Cystic lesions with a diameter over 10 mm, or less than 10 mm but suspicious for malignancy (papilliferous proliferations etc.), were also punctured and aspirated. 

All patients had their first UG-FNAB. Patients with subsequent UG-FNAB due to inconclusive specimens were not included in this study population. All patients signed an informed consent before the procedure. 

In this study, all FNAB were ultrasound-guided (the different techniques for UG-FNAB are reviewed in [21, 22]) and a linear transducer 7.5 MHz (Fukuda 5500, Japan) was used. Patients received no anesthetic and were in the standard supine position during the intervention. We used a “free hand” technique with a parallel approach, needles of 20 and 22 G and syringes of 10 cc. Almost all of the nodules (except the smallest) were punctured at least twice. We only used nonaspiration (capillary) biopsy in a few cases (8%) when the aspirate in two consecutive punctures of one and the same nodule was obviously blood. The smears were dried for 24 hours at room temperature and then processed with Pappenheim staining. Cytological analysis was routinely performed by a specialist experienced in thyroid pathology. For the purposes of this analysis the findings were divided into 5 main groups: malignant, adenoma, MNG, inconclusive, and others. Moreover, the ultrasound category MNG and its cytological presentation were split in “benign” and “malignant” subcategories. Further, malignant lesions were differentiated into different cytological subgroups (Table 1). Other parallel findings were also recorded, but are not an object of this particular analysis.

The comparison between ultrasonography (U) and cytology (C) findings was made as follows. The results obtained by both methods were divided into malignant (including “probably malignant”, “suspicious for malignancy” etc.) = positive (U+ and C+) and most likely nonmalignant = negative (U− and C−). Further, we considered U+C+ as true positive (TP), U+C− as false positive (FP), U−C− as true negative (TN) and U−C+ as false negative (FN). We calculated the sensitivity = TP/(TP+FN), specificity = TN/(TN+FP), positive predictive value = TP/(TP+FP), negative predictive value = TN/(TN+FN), and diagnostic accuracy = (TP+TN)/(FP+FN+TP+TN). Patients with two different findings in different nodules were given a special category. The main question that this invasive diagnostic method is designed to answer is “malignant or not?”. We considered the US diagnoses (adenoma/multinodular goiter or probably malignant) that corresponded to the diagnoses from cytology to be coincidental, independent of the additional cytological finding. For example, a patient with 3 nodules and US diagnosis, that is, multinodular goiter, was interpreted as coincidental, but if one of these 3 nodules was malignant, we considered this was not coincidental with the cytology. The terms adenomatous goiter, nontoxic nodular goiter, and colloid nodular goiter are used interchangeably as descriptive terms when a multinodular goiter is found [23].

Where possible, the comparison between cytology (C) and final histology (H) results was calculated in the same way in those patients who had been operated on. If records in our registry were lacking, we tried to contact all of the C+ patients to obtain information if his/her thyroid had been operated and to analyze the histological results. We were unable to obtain data for these comparisons in most of the outpatients with negative cytology results, because this was a retrospective study performed in an endocrine department, and these patients were not operated on and lost to followup.

### 2.1. Statistical Methods

Exploratory analysis was performed including graphical presentation of distribution and usual descriptive statistics. Difference between two proportions tests were applied for sensitivity, specificity, positive and negative predictive values, diagnostic accuracy and C+malignancy rate. Differences between solitary and MNG cases were tested. The *P*-level values ware computed based on the *t*-values for the respective statistics. Both one-sided and two-sided tests were performed. The differences were considered significant at *P* < 0.05. The computations were performed by STATISTICA (data analysis software system StatSoft, Inc., version 5.5).

## 3. Results

**Figure 1:**
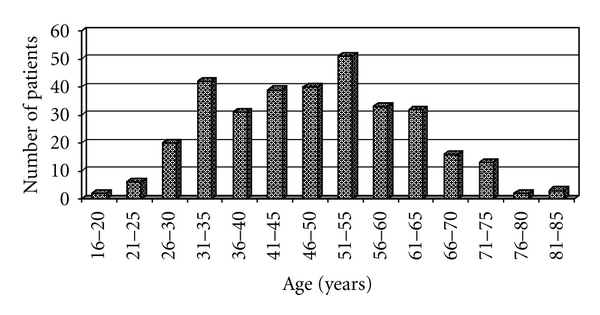
Age distribution of the patients.

From the 359 patient records studied 29 were excluded because of insufficient information in the US-exam records. The remaining 330 patients included 306 women and 24 men (12.8 : 1). The mean age ± SD was 48.4 ± 11.2 years ranging from 16 to 84 years (Figure 1). 

A total of 596 nodules were found by ultrasound. UG-FNAB was performed on 546 nodules (91.6%). In most cases of MNG, only the three biggest nodules were punctured. The total number of punctures was 1231, representing 2.3 punctures per nodule and 3.7 punctures per patient.

**Figure 2:**
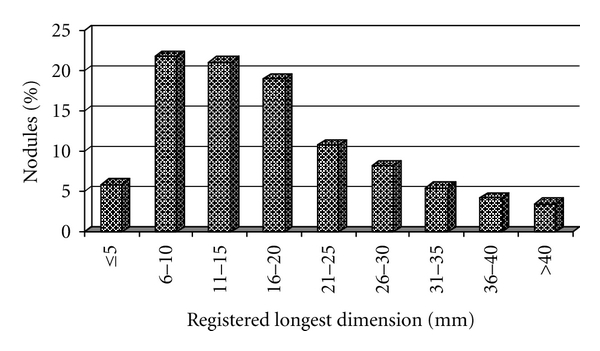
Distribution by size of the punctured nodules.

The volume of the nodule could not be calculated from all records, and we used the longest registered diameter for characterization of the nodular size (Figure 2).

**Figure 3:**
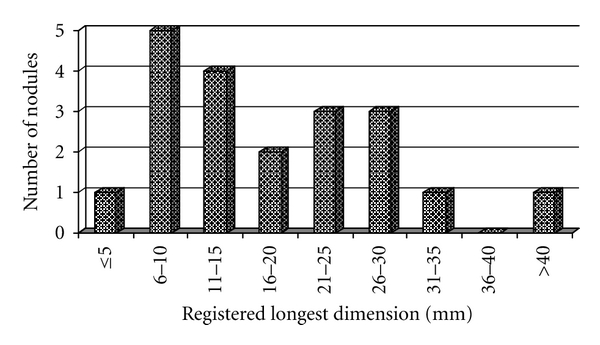
Distribution by size of the malignant nodules.

From the 330 patients with 546 punctured nodules 16 patients (4.8%) with 18 nodules were excluded after cytology because of noninformative results. Another 7 patients (2.1%) with 16 pseudonodules (T. Hashimoto as sole diagnosis) were also excluded. This left data from 307 patients with 512 nodules (Table 2) for further analysis. In this group, 54 nodules in 50 patients were evaluated by US as “probably malignant”, but this suspicion was only confirmed by cytology for 14 nodules in 12 patients. Six more cancers were found in 6 patients who were U−. In total, 20 nodules were malignant in 18 patients by cytology. One of these patients (U+ with MNG) had bifocal carcinoma, and another (U+ with MNG) had two metastases from breast cancer. The percent of carcinomas diagnosed by cytology in this study was 3.9% of nodules and 5.9% of patients (Table 1 and Figure 3). Two of these 18 patients were lost to followup after the UG-FNAB. The cytology : histology coincidence analysis was conducted on the data from the remaining 16 patients. From their 18 C+ nodules, 15 (83.3%) were confirmed after surgery, but 3 were not. In 13 out of the 16 C+ patients the diagnosis was verified as H+. 

The subgroup of patients with solitary nodules comprised 151 patients with 151 nodules. Of these 22 were U+ but only 8 were C+. From the 129 U− nodules 2 were C+. One patient was lost to followup. The histology confirmed the diagnosis in 8 of these 9 C+ nodules (88.9%).

156 patients had MNG with a total number of 361 punctured nodules. 28 patients were U+ with 32 suspicious nodules and 4 of them were C+ with 6 nodules. Missed by ultrasound were 4 C+ patients and two of the malignant nodules were nondominant. One patient was lost to followup and from the 7 C+ patients with 9 nodules histology confirmed malignancy in 5 with 7 nodules (71.4% of the patients and 77.8% of the nodules) (Table 2).

The ultrasound/cytology coincidence analyses indicated a lower positive predictive value and diagnostic accuracy (*P* < 0.05) and a borderline difference in specificity (*P* = 0.066) for patients with solitary nodules compared to MNG.

None of the cancers was found in a pure cyst. Only one of the cancers (papillary) was hyperechoic and only one was less than 5 mm. Other parallel findings (37 cases) included 28 thyreoiditis Hashimoto, 5 thyreoiditis subacuta, 1 intrathyroid parathyroid adenoma, 2 candida inflammatory changes, and 1 sarcoidosis.

UG-FNAB is a safe diagnostic method. We had to stop the procedure twice because of a hypotensive reaction, but no additional medication or reanimation measures were necessary in these cases. The patients knew from the informed consent form that they could experience some pain after biopsy and common analgesics are appropriate drugs in such cases. Only two patients complained of pain after the manipulation, one of whom had a substantial subcapsular hematoma. No additional surgical procedures were necessary.

## 4. Discussion

The superficial position of the thyroid is an advantage compared to other endocrine glands and FNAB has become a simple and reliable method for thyroid evaluation. Most studies with FNAB have not used US guidance. Larger nodules have tended to be detected because usually palpable nodules are bigger than 10 mm, [23]. The introduction of real-time ultrasound guidance has greatly improved the sensitivity and specificity of FNAB [18]. In palpable nodules, biopsies can be obtained with more precision (1) exactly from the targeted nodule and (2) from its more-informative zones, which are usually peripheral, rather than central and often necrotic areas. In heterogeneous nodules, biopsies should be taken from the hypoechoic area of the nodule and in cysts or mixed nodules they should be from the wall and areas with papilliferous proliferation [21]. The “nondiagnostic” rate decreased from 15%–20% in earlier FNAB studies to around 3% in studies using UG-FNAB [17, 24]. This decrease is important in assessing the costeffectiveness of the method. Some investigators report an even higher effectiveness of UG-FNAB with only 0.7% unsatisfactory biopsies [25]. Our study results were inconclusive for 4.8% of the patients and 3.3% of the nodules. Although not the prime aim of this study, we analyzed the fate of these patients with inconclusive first biopsies in a separate series. Of the 16 patients 10 underwent a secondary UG-FNAB which was sufficient for diagnoses in 9 (90%), but no cancer was found. Some studies have reported no further increase in diagnostic power of secondary biopsy [26]. Bearing in mind the low failure rate in primary UG-FNAB and the low probability of failure on repetition we recommend rebiopsy in such cases.

The most important advantage of UG-FNAB over FNAB is the possibility of puncturing nonpalpable nodules (size 10–15 mm or less). There is no consensus on whether it is necessary to biopsy such nodules (incidentalomas). Contra-arguments are that they are very common, their prognosis, even in the case of malignancy, is not so poor, and routine biopsy is not costeffective. Proarguments are that the incidence of malignancy is the same in palpable and nonpalpable nodules [27, 28] and such small carcinomas are often as aggressive as larger ones [29]. In our study, 42.9 % of the nodules were between 5 and 15 mm in their greatest dimension, and 9 carcinomas were found in this group, which is 45% of all malignancies diagnosed through cytology in this study. In nodules less than 5 mm (5.9% of all nodules), we found 1 cancer (5% of the malignant nodules). The rate of cancers in this subgroup was the same as in the whole group. Our impression is that nodules in the range 5–15 mm should be punctured because of their high malignancy potential and the improved prognosis after surgical treatment. We are especially rigorous in screening young patients and puncture every solid or mixed nodule found. We adopt this approach also for nodules less than 5 mm. A physician inexperienced in puncturing such nodules would be advised to follow up regularly. Most of the nodules found in this study were less than 2 cm, but this could be explained by patients with larger lesions being directly referred to surgery, according to the old rule “If a nodule is greater than 2 cm it should be removed!”.

In our study, every patient who had at least one nodule underwent UG-FNAB and a high proportion (91.6%) of all nodules were punctured. Other specialists use narrower indications for biopsy, but most agree that ultrasound is not reliable enough for a solid nodule to be classified as benign or malignant. In total, 20 carcinomas were found (3.9% of the nodules) in our series and half of them were in solitary nodules, which are a proper indication for UG-FNAB [30, 31]. 

The decision to biopsy a MNG is more complex. The incidence of malignancy in multinodular goitre ranges from 1% to 10% [32–35]. One study reported the sensitivity of FNAB in MNG at only 17%, but the specificity at 96%, diagnostic accuracy at 88%, the positive predictive value at 32% and the negative predictive value at 88% [36]. The authors concluded that FNAB is not useful for differentiating MNG with malignant degeneration from benign MNG because more than 80% of carcinomas go unnoticed, and they suggested that clinical criteria should prevail over FNAB. Although most of the biopsies were not ultrasound guided the study raises an important question for the diagnostic value of this method in MNG. Our study confirmed lower positive predictive value, diagnostic accuracy and a borderline difference in specificity for patients with solitary nodules compared to MNG.

 In our study, a specific separate approach for nodules and patients was used. We found malignancy in 10 of the nodules in MNG (2.7%). This is about 2.4 times lower than that we found in solitary nodules (6.6%), but this low malignancy risk for each nodule is multiplied by the higher number of nodules. The resulting risk for the particular patient, as shown in this study, does not differ substantially from the risk if a solitary nodule presents. 

As mentioned in “Patients and Methods”, the lack of histological control for most of our C-patients is a serious limitation of our study. But this is usual in an endocrinology unit where referral of a C-patient for surgical exploration cannot be justified. The ultrasound monitoring of these patients is only a partial solution to the problem, especially for small nodules, considering the slow natural evolution of such benign, and some malignant, lesions as well as the “adherence to the physician” factor. Core biopsy is another way of improving the diagnostic value of this method, but it is more expensive and still not as popular as conventional UG-FNAB. Another possibility is to biopsy most of the nodules in a MNG and to obtain more samples for each nodule (2.3 in this study). The reasoning and considerations for such an approach are the following.

1. Although very probable, carcinomas are not always located in the dominant nodule, which is punctured most often. Taking samples from other nodules increases the chances of diagnosing an occult malignant process. In our study, two of the malignant nodules in MNG were not suspected from ultrasonography and were nondominant.
2. In some cases, the US and surgical reports are not topographically consistent, and it is uncertain if a particular nodule that has or has been not punctured is the malignant nodule found by surgery. Such artificial lack of coincidence could interfere with the statistical evaluation indices.
3. Perioperative complications, postoperative state and treatment indications, and the probability of relapse are also arguments to be considered.
4. The final decision for regular US monitoring, UG-FNAB of the dominant nodule, multinodule, and multipuncture UG-FNAB or surgical exploration, especially in a patient whose MNG is not obviously suspicious for malignancy and/or very large, is an economic one. UG-FNAB and US followup is less expensive than surgery. Measurement of each nodule on every consecutive visit is not always accurate enough and is time consuming. If a biopsy has been performed, the interval between the visits could be longer, saving time and money of the health care system, without decreasing the medical vigilance. The endemic aspects of MNG should be taken into consideration as well.

In our series, no cancer was found in a pure cyst and only one was hyperechoic. We agree with the conclusion drawn by Leenhardt et al. [37], who analysed 450 nonpalpable nodules, that cystic and hyperechoic nodules are not indications for UG-FNAB (nevertheless cysts are often punctured and aspirated for diagnostic and/or treatment reasons). In patients with more than one nodule only the FNAB on the largest one was retained in their study. They estimated a cytological malignancy of 5%, suspicious 11% and after excluding the patients lost to followup a histological malignancy rate of 4%. Bearing in mind the high percentage of unsuspected cancers in nondominant nodules, we suggest that most nodules should be punctured in a MNG. 

The rate of the carcinomas in our study is less than that reported by others. The reasons for this could be the unselected population. Almost all nodules were punctured. In most other studies, especially those originating from surgical or morphological departments, nodules that are punctured are the preselected ones found suspicious at the clinical and US level, which increases the cancer probability. 

## 5. Conclusions

UG-FNAB is a safe and reliable diagnostic approach for thyroid nodules. It is the method of choice for hypo- and isoechoic not purely cystic solitary nodules, regardless of the nodule size. In MNG, its positive predictive value and diagnostic accuracy are lower. The final decision for regular US monitoring, UG-FNAB of the dominant nodule, multipuncture UG-FNAB or surgical exploration is one of complex appraisal. We consider UG-FNAB appropriate for most nodules in MNG according to the above-mentioned criteria.

## Figures and Tables

**Table 1:** Malignant nodules, detected by cytology.

Cytology	*N* of patients
Papillary	8
Papillary (follicular variant)	1
Follicular	3
Follicular-Hurtelcellular	1
Hurtelcellular	2
Undifferentiated	1
Atypical parafollicular	1
Metastatic (breast)	1

Total	18

**Table 2:** Comparison between ultrasound, cytology, and histology results.

		Nodules			Patients	
	Total	Solitary	MNG	Total	Solitary	MNG
*N* =	512	151	361	307	151	156
U+C+	14	8	6	12	8	4
U+C−	40	14	26	38	14	24
U−C+	6	2	4	6	2	4
U+C−	452	127	325	251	127	124
Sensitivity (%)	70.0	80.0	60.0	66.7	80.0	50.0
Specificity (%)	91.9	90.1	92.6	86.9	90.1	83.8
Positive predictive value (%)	25.9	36.4	18.8	24.0	36.4	14.3*
Negative predictive value (%)	98.7	98.4	98.8	97.7	98.4	96.9
Diagnostic accuracy (%)	91.0	89.4	91.7	85.7	89.4	82.1*
C+ malignancy rate (%)	3.9	6.6	2.7	5.9	6.6	5.1

C+H+	15	8	7	13	8	5
C+H−	3	1	2	3	1	2
Positive predictive value (%)	83.3	88.9	77.8	81.3	88.9	71.4

*P* < 0.05 between patients with solitary nodules and MNG.

U+ and U− ultrasound positive and negative.

C+ and C− cytology positive and negative.

H+ and H− histology positive and negative.
